# Barbs Facilitate the Helical Penetration of Honeybee (*Apis mellifera ligustica*) Stingers

**DOI:** 10.1371/journal.pone.0103823

**Published:** 2014-08-04

**Authors:** Jianing Wu, Shaoze Yan, Jieliang Zhao, Yuying Ye

**Affiliations:** Division of Intelligent and Biomechanical Systems, State Key Laboratory of Tribology, Department of Mechanical Engineering, Tsinghua University, Beijing, P. R. China; University of Heidelberg Medical School, Germany

## Abstract

The stinger is a very small and efficient device that allows honeybees to perform two main physiological activities: repelling enemies and laying eggs for reproduction. In this study, we explored the specific characteristics of stinger penetration, where we focused on its movements and the effects of it microstructure. The stingers of Italian honeybees (*Apis mellifera ligustica*) were grouped and fixed onto four types of cubic substrates, before pressing into different substrates. The morphological characteristics of the stinger cross-sections were analyzed before and after penetration by microscopy. Our findings suggest that the honeybee stinger undergoes helical and clockwise rotation during penetration. We also found that the helical penetration of the stinger is associated directly with the spiral distribution of the barbs, thereby confirming that stinger penetration involves an advanced microstructure rather than a simple needle-like apparatus. These results provide new insights into the mechanism of honeybee stinger penetration.

## Introduction

In variable and complex environments, animals are equipped with different organs for accomplishing diverse physical activities. Stingers and needles are found in some insects in the orders Diptera and Hymenoptera, where they play important roles in predation, mating, and defense [1]–[5]. Various theories have been developed to describe the penetration mechanism of insect stingers and needles [6]–[9]. A comprehensive understanding of stinger penetration has been obtained gradually, which has attracted the interest of the developers of bio-inspired instruments, e.g., painless insertion for medical care [10] and bionic-based drilling technologies for planetary subsurface exploration [11].

**Figure 1:**
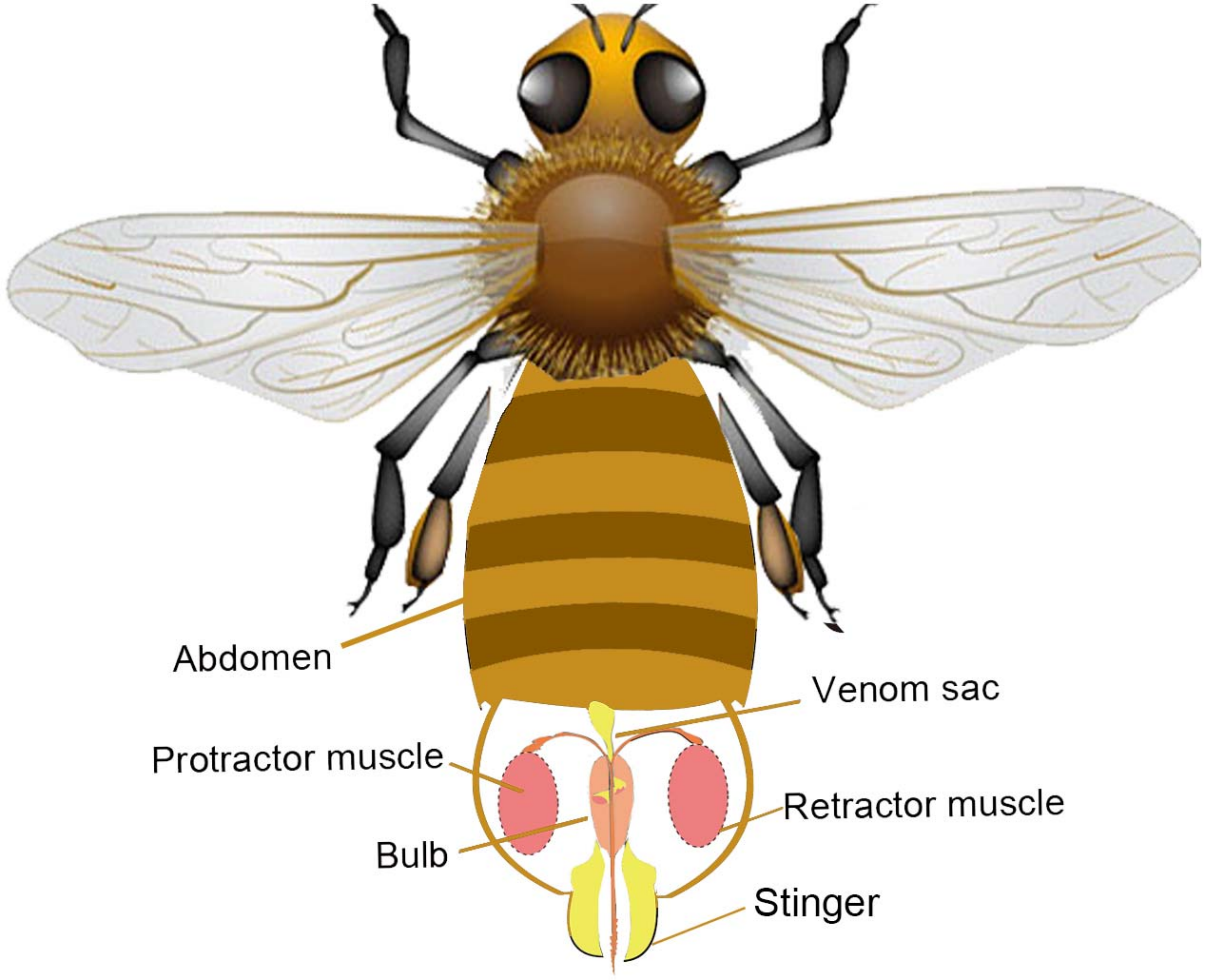
Anatomy of the honeybee’s stinger apparatus. The stinger resides in the sting chamber inside the last abdominal segment (not to scale). The sting apparatus mainly comprises the protractor/retractor muscles, the bulb, the stinger, and the venom sac. The protractor muscles drive the stinger to penetrate the wound and the retractor muscles are used in the reverse manner to pull the stinger back into the sting chamber. During penetration, the venom is pumped into the stinger from the bulb, which is also known as the venom reservoir.

The abdomen of the honeybee (*Apis mellifera*) comprises 10 segments, seven of which are obvious [12]–[14]. The cavity within the last abdominal segment of the honeybee is called the sting chamber and the entire sting apparatus is enclosed within the chamber when it is not in use, as well as nerve ganglions, various muscles, a venom sac, and the end of the insect’s digestive tract [15]–[16]. The stinger is a small and delicate device, which allows honeybee workers to defend their nest against predators [12]. As shown in Fig. 1, when dangerous enemies are encountered, the sting apparatus receives a signal from the nerve ganglions and the bee bends its abdomen downward due to muscle contractions as it prepares for vertical stinger penetration. During the use of the stinger, two pairs of protractor and retractor muscles move the stinger up and down, which causes a flexible extension of the stinger shaft. Movements of the bee’s legs, the muscles of the abdomen, and the effect of the backward pointing barbs combine to produce a thrust that drives the stinger efficiently, and the venom is delivered instantly into tough skin through a channel in the stinger. The first analysis of the stinger penetration mechanism was performed by Dade in 1890s, particularly the coordination between various organs [14].

The stinger comprises two lancets with groups of curved barbs on the outer aspects of their distal ends, which are held in grooves on the stylet [14]. It is well known that the main role of the barb is to provide one-way traction, which allows the stinger to work itself deeper into the flesh [13], [14]. The raked structure of the barbs makes it difficult to remove the sting, which might help the bee to continue pumping venom into the flesh via the detached stinger for a relatively long time [14]. In this case, the underlying mechanism of penetration appears relatively simple, i.e., the needle-like stinger is assumed to move axially while piercing the skin, but the possible role of rotation along the stinger shaft has been neglected. This is because the bee stingers measure a few millimeters and the action of stinging occurs within one second, thus the actual penetration behavior cannot be observed easily. Previous research has only considered the morphology at the level of a single barb. However, the potential effects of the distribution of the barbs on the efficiency of penetration have not been identified clearly.

In this study, we explored the penetration mechanism of the honeybee stinger. We investigated the morphology of the barbs on the bee stinger and elucidated the specific factors that determine the rotation of the stinger. Our results showed that the stinger undergoes helical and clockwise rotation during penetration, where the spiral distribution of the barbs is responsible for this phenomenon.

## Materials and Methods

### Experimental method

**Figure 2:**
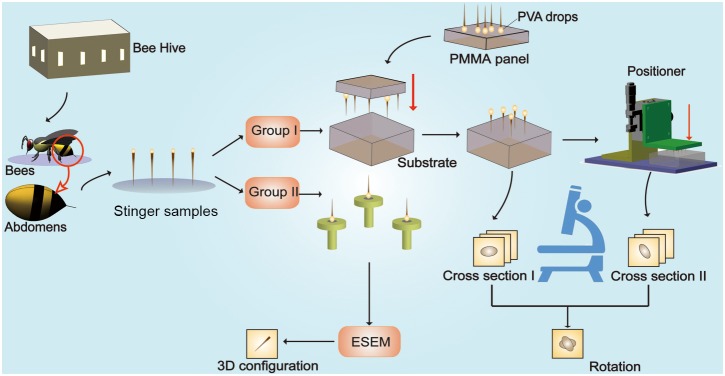
Preparation of the stinger samples and the experiments. The stingers of worker bees were collected and separated into two groups. (1) The first group of stingers were placed onto the polymethyl methacrylate panel using drops of 15% polyvinyl alcohol (0.1 µL), and they were then placed vertically on the substrates (agar, silica gel, soft rubber and paraffin wax), before pushing the stingers into the substrates at a velocity of 6 mm mm/s using the positioner. The positioner, also called the precision position platform, is a machine that is able to push tiny appendages accurately into the substrates following the planned kinematics, for instance the preset average velocity, the total displacement even the acceleration. (2) The microstructures of the second group of stingers were observed using an environmental scanning electron microscope.

We studied the penetration characteristics of the stingers of honeybee (*Apis mellifera ligustica*) workers. The samples were collected at Tsinghua University of Beijing, China (40.000153°N, 116.326414°E). No specific permissions were required for these locations/activities. We confirm that the field studies did not involve endangered or protected species. To ensure the reliability and repeatability of the experiments, all of the honeybee samples were captured around wild bee nests and the experiments were conducted within 1 h of collection. In total, 30 fresh stingers from worker bees were selected, cleaned, and dehydrated, where the average length was 8.5 mm (Fig. 2).

We performed two types of experiments to elucidate the stinging mechanism. As shown in Fig. 1, we first separated the stingers into two groups, where group I and group II contained 20 and 10 stinger samples, respectively. The 20 stingers in group I were then grouped into subgroups A_1_, A_2_, A_3_, and A_4_, each of which comprised five samples. Four types of 10×10×10 mm cubic substrates were prepared, which were made of agar, silica gel, soft rubber, and paraffin wax. The samples in Groups A_1_∼A_4_ were fixed to the 20% *Poly Vinyl Alcohol* (PVA) colloid droplets which were firstly dispensed on the PMMA panel. Thereby tips of the stings were placed onto the substrate of the cubic block of different materials (See Figure S3 in File S1). All of the stings were pressed for 6.0 mm into the substrate with a precision position platform with the average velocity approximately to 6 mm/s (Fig. 2). In addition, to eliminate any errors caused by the setup, we tested rotation angles of the human hair samples that measured ca 8.5 mm in length for comparison. Morphological images of cross-sections of the stingers and hairs were obtained before and after penetration by microscopy. Notably we observed the natural heads of the stingers directly to determine whether the helical penetration exists or not [17]. With the help of the environmental scanning electron microscope (ESEM), we observed the microstructure of 10 stingers in group I.

### Rotation measurement

**Figure 3:**
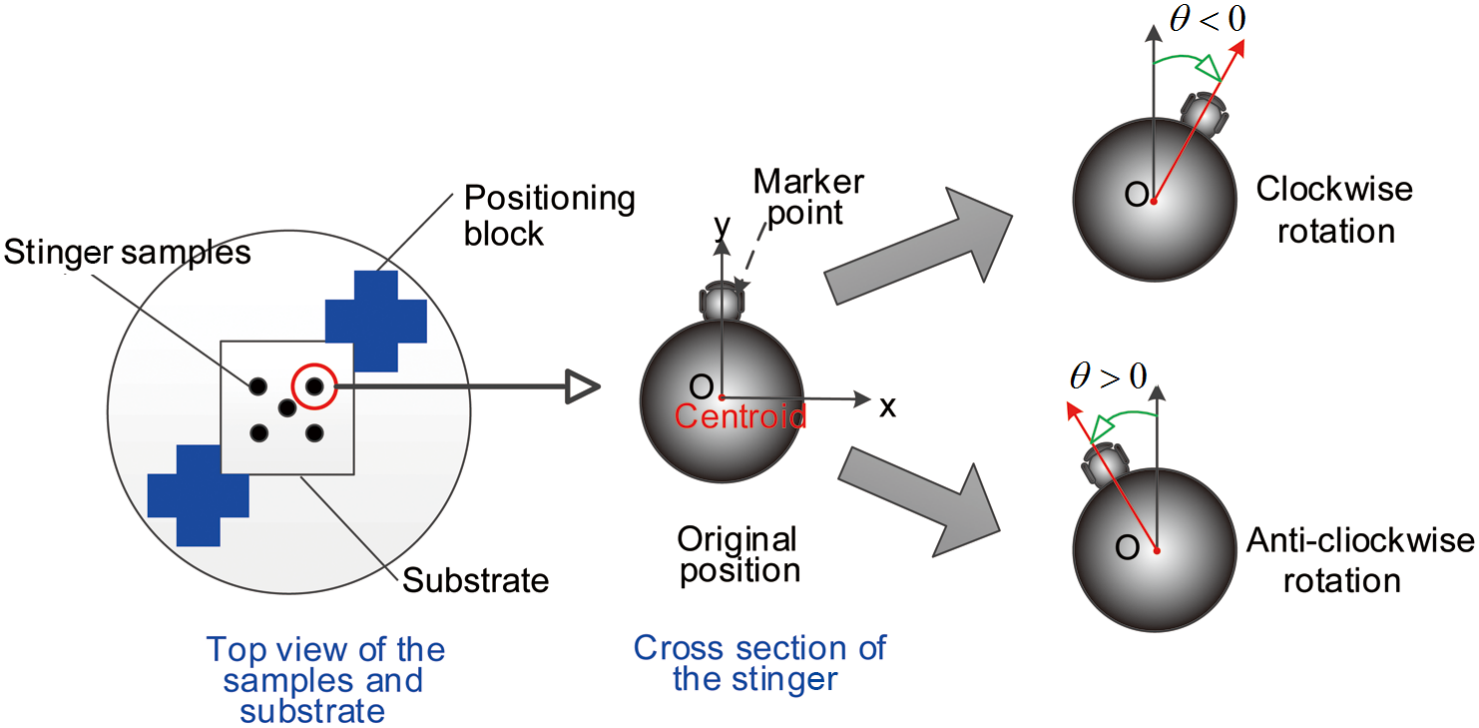
Observation of the stinger cross-sections and calculation of the rotation angles. (A) The four types of cubic substrates were attached to the positioning block assembly under the microscope. (B) Marker points in the stinger cross-section. If the stinger rotated clockwise, the rotation angle was recorded as a negative angle, whereas an anticlockwise rotation was recorded as a positive angle.

Fig. 3 shows the method used to observe the stinger cross-sections and to calculate the rotation angles. The cross-sections of the stinger were observed and photographed before and after penetration. We enhanced the microscope so it could locate the cubic substrates by using a positioning block, thereby ensuring that the cubic substrate remained fixed. The rotation angles were measured by comparing the positions of markers in the stinger cross-sections.

The image processing system used to capture the contours of the cross-sections of the stingers was implemented with the Canny operator. We define the equation of the contours as 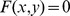, thus the centroid of the cross-section, which is denoted as *O* (*x_O_*, *y_O_*), can be calculated by
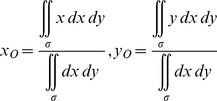
(1)where 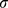 represents the region surrounded by the contour of the stinger shaft. The key point used to measure the change in configuration is defined as 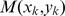, which satisfies



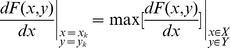
(2)We define the coordinates of the key points before and after penetration as 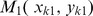 and 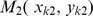, respectively. By comparing the contours of the cross-sections, we determined the amount of rotation before and after penetration. In this case, the rotation angle is 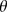


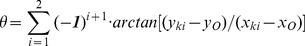
(3)where 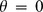
indicates that the stinger shaft does not rotate, whereas 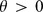 and 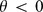 indicates that the stinger shaft rotates in clockwise or anticlockwise directions, respectively.

As stated above, the remaining 10 stingers were used to obtain microstructural observations with an ESEM. Particular emphasis was placed on the physical distribution and geometrical properties of the barbs on the stinger.

## Results

### Helical penetration

**Figure 4:**
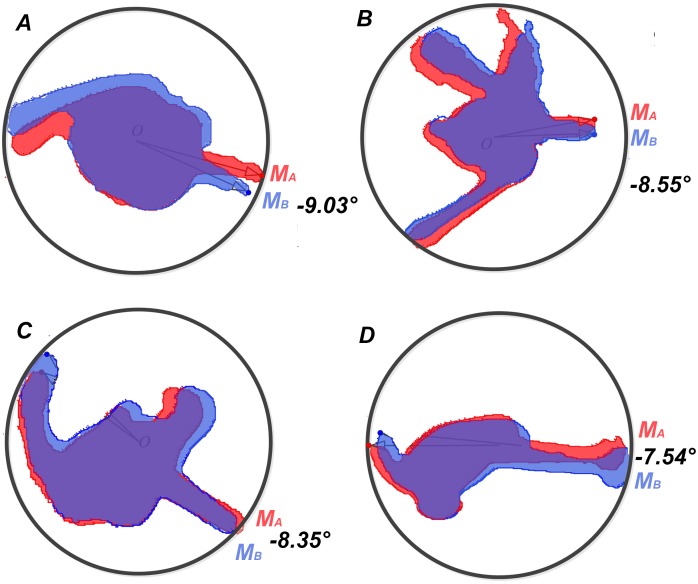
Cross-sections of the stinger shafts. We determined the outlines of the stinger cross-sections by applying the Canny operator. (A)–(D) show the four stinger cross-sections before and after penetration of the substrates, i.e., agar, silica gel, soft rubber, and paraffin wax, respectively. Helical penetration was identified clearly by analyzing the rotation angles of the marker points. We also found that the rotation angle decreased when we used relatively harder substrates.

**Figure 5:**
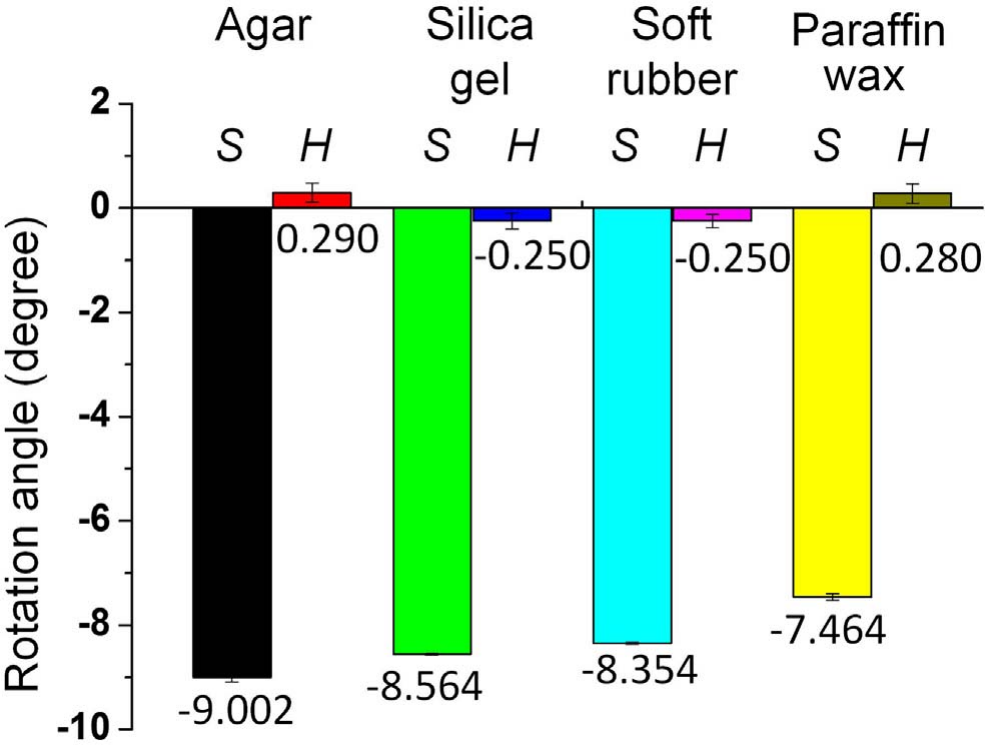
Rotation angles of the stinger shafts. The stinger samples marked with *S* and the hair samples marked with *H* were used for comparison. The two adjacent bars show the rotation angles of the honeybee stingers (left side) and the hair samples (right side). In each type of substrate, the rotation angle of the hair was very small, which demonstrated that the instrument had no significant effect on the rotation angle during pushing. The experimental observations confirmed the existence of rotation during the stinging process. Furthermore, we found that the rotation angle was associated with the stiffness of the substrate.

Fig. 4 shows four cross-sections of stingers that penetrated the agar, silica gel, soft rubber, and paraffin wax substrates. The features of helical penetration are shown in the comparison chart. In particular, *M*_A_ and *M*_B_ are the marker points that indicate the rotation angles according to Eqs (1)–(3). We averaged the rotation angles for the subgroups A_1_, A_2_, A_3_, and A_4_ which were collected from the experiments, and calculated the standard deviations of the data in different groups to test the data stability. Statistical analysis demonstrated that the data were stable with the standard deviation around 0.046° (See Table S1 in File S1). By comparing the stinger sections before and after penetration, we found that the mean rotation angles of the 20 samples were all negative, which showed that the stingers rotated in a clockwise direction while penetrating the substrates (Fig. 5). By contrast, the rotation angle of the hair section was <0.3°, which demonstrated that the experimental setup did not introduce large errors, thereby validating the results. As shown in Fig. 5, the average rotation angle of the stinger shaft based on all the data was –8.364°. The rotation angle decreased gradually as the hardness of the substrate surface increased, i.e., from the agar and silica gel, to the soft rubber and paraffin wax. The hardness of the silica gel is closest to the human skin, indicating that the stinger may approximately rotate –8.0° when it penetrates the human skin.

### Microstructure of the honeybee stinger

**Figure 6:**
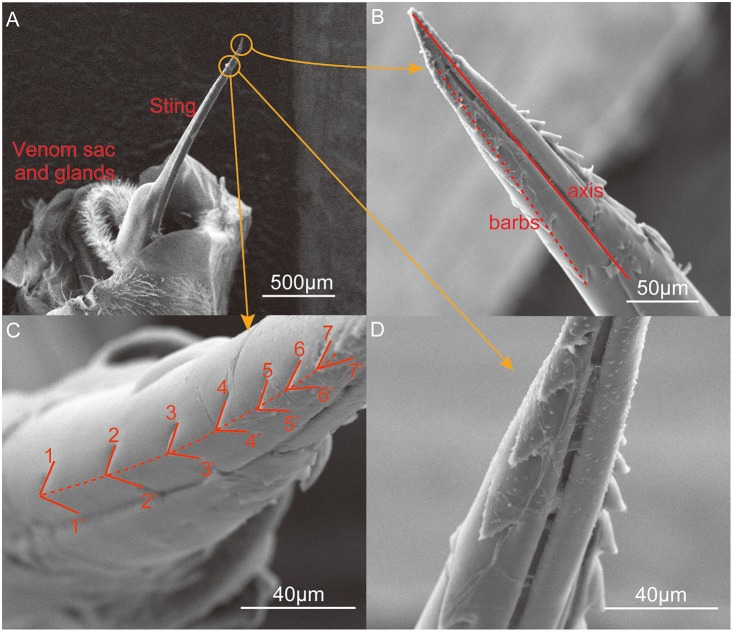
Environmental scanning electron microscope images of the stinger. (A) The needle-like sting, venom sac, and related glands. The stinger is activated by the muscles to penetrate the skin of the victim. (B) Barbs along the axial direction of the sting. The solid line in Figure 3(b) is the axis of the sting which is obtained by connecting the tip of the stinger and the midpoint of the stinger root. The stinger of *Apis mellifera ligustica* has two rows of barbs, each of which comprises about 10 barbs. The angle between the rows of barbs and the axis of the stinger shaft was around 8–9°, according to observations based on 10 samples. The row of barbs was found to form a right-handed helix. (C) Magnified view of the barbs. Seven barbs are marked with the notations 1–1′, 2–2′, etc. Note that the angles of the tips were 90.33°, 89.62°, 80.31°, 72.13°, 72.36°, 59.63°, and 46.19°, thereby demonstrating that the barbs were relatively sharper near the tip of the stinger. (D) Magnified view of two rows of barbs. Viewed in the axial direction, the angles between the two rows of barbs were about 95°.

This rotation may be attributable to the shape of the stinger and the distribution of the barbs. Understanding the underlying mechanism will help to elucidate the stinging process and related behaviors. Fig. 6 shows the microstructure of the stinger, which was obtained by ESEM. The venom sac and related glands support the stinger. The bee stinger is generally similar to a long needle and its proximal apex is covered by two lines of barbs, which subtend two angles with the stinger axis. The tetrahedron-shaped barbs decrease in size as they approach the tip of the stinger.

## Discussion

Our results showed that the stinger shaft rotates when it is pushed into a solid substrate and its rotation angle might be affected by the hardness of the substrate. Thus, it is possible that the helical penetration might improve the puncture process, in a similar manner to the spiral flight of bullets. In addition, this specific penetration behavior increases the difficulty of sting removal, which might increase the amount of venom injected. To better understand the mechanism of the rotation of the stinger shaft, it is important to explore the dynamic performance during penetration. In particular, it is possible that the microstructure of the stinger facilitates the rotation of the stinger shaft, especially the protuberances.

**Figure 7:**
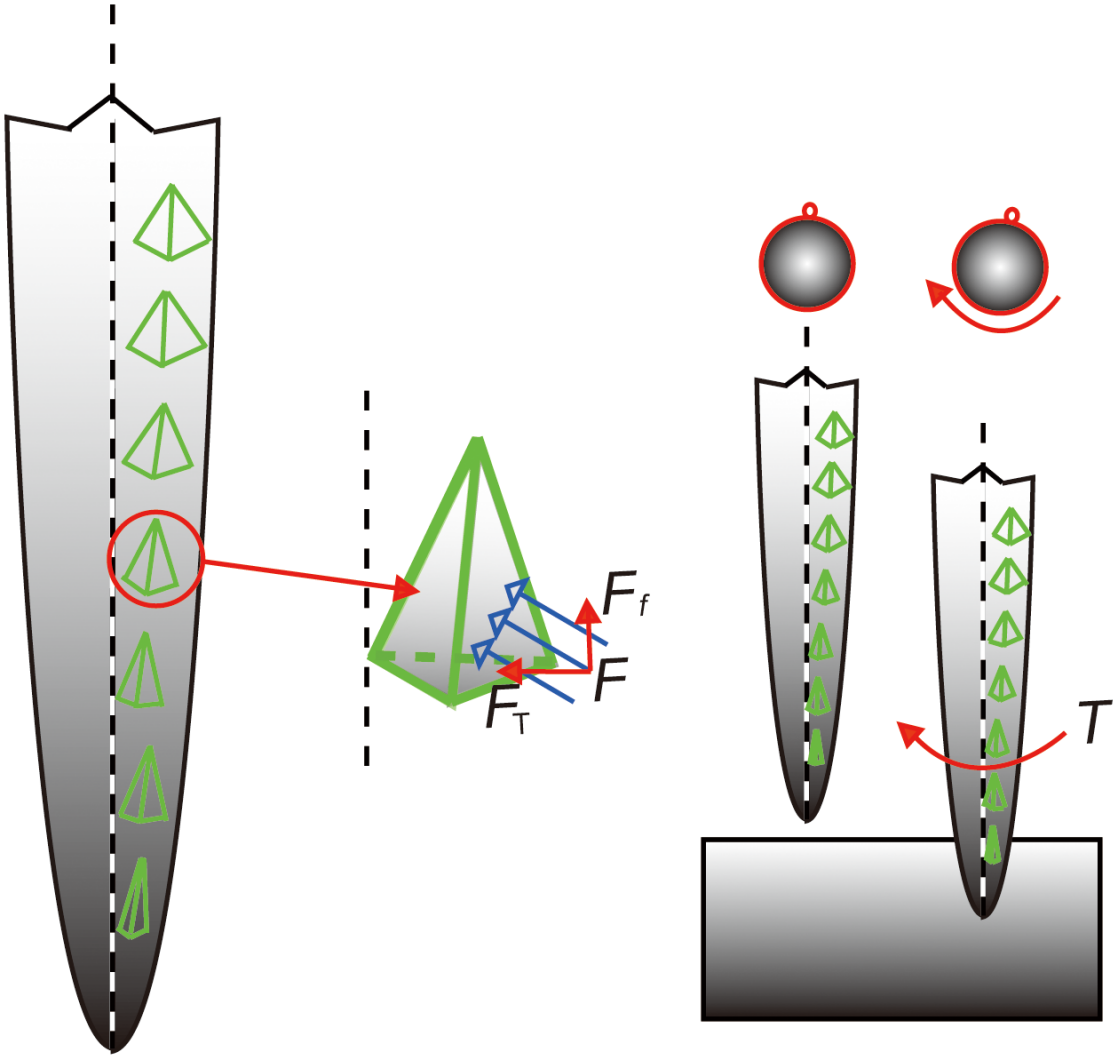
Mechanistic model of stinging. The angle between a row of barbs and the shaft axis was about 9°. The equivalent tetrahedron model mimics the force condition of a single barb (not to scale). During penetration, the contact force on the barb (*F*) can be decomposed into the friction force (*F_f_*) and the tangential force (*F*_T_). The torque around the stinger shaft is generated by the tangential force, which drives the stinger to rotate clockwise in the line of sight along the stinger shaft toward the tip.

Fig. 7 shows a mechanistic model of stinging that considers the microstructure of the barbs. The tetrahedron model mimics the force condition of a single barb. During penetration, the contact force is not parallel to the stinger axis, therefore the torque generated by the tangential force (marked as *F*_T_ in Fig. 7) can be calculated by *T* = *rF*_T_, where *r* is the corresponding radius of the stinger. By focusing on the right-handed helical distribution of barbs, the angle between the line of barbs and the stinger shaft is about 8.523°, which agrees with the average rotation angle (–8.364°) shown in Fig. 5. The blunter barbs located further from the tip of stinger will expand the wound size during penetration. Therefore, the force analysis demonstrates that the helical distribution of barbs leads to the rotation of the stinger during penetration.

The specific microstructure of the stinger provides new insights, which may facilitate a deeper investigation of the biological significance of stinger penetration. The main appendages of the bee stinger of *Apis mellifera ligustica* are derived from the segmental appendages of the 9^th^ and 10^th^ abdominal segments. The appendages of all segments are organized into segments and branches during the early stages of evolution beyond the primitive worm stage, according to the same plan, after which they have been modified in different ways for specific purposes [14], [18]. According to many researchers [16], the worker bees represent the body organs required for nest maintenance, digestion, and defense [15], [19]–[20]. Helical penetration might improve the effectiveness of attack with fewer sacrifices. The development of novel devices may also be inspired by this specific penetration mechanism [21], [22].

## Supporting Information

File S1 **Contains the files: Figure S1.** Principle of experiment. **Figure S2.** Samples of the worker bees’ stings. **Figure S3.** The precision positioner and the substrate. **Figure S4**. Location of the substrate. **Figure S5.** Morphology of the cross section. **Figure S6.** Rotation angles of the sting shaft. **Table S1.** The experimental results from the observation of the rotational angles.
(DOC)
