# Supplementary material for: Barbs Facilitate the Helical Penetration of Honeybee (Apis mellifera ligustica) Stingers
Source: PLoS One. 2014 Aug 4;9(8):e103823. doi: 10.1371/journal.pone.0103823 (PMC4121201; doi:10.1371/journal.pone.0103823)
Supplement: File S1 — Contains the files: Figure S1. Principle of experiment. Figure S2. Samples of the worker bees’ stings. Figure S3. The precision positioner and the substrate. Figure S4. Location of the substrate. Figure S5. Morphology of the cross section. Figure S6. Rotation angles of the sting shaft. Table S1. The experimental results from the observation of the rotational angles. (DOC) [file pone.0103823.s001.doc]

**SUPPORT LETTER**

1. **Main principle of the experiments**

We made 4 cubic substrates out of agar, paraffin, soft rubber and silica gel. Stings were perpendicularly put on each substrate (5 sting samples per one substrate) and pushed 6*mm* into the substrate with a precision position platform. Figure S1 shows the principle of the experiments.

| (A) (B)  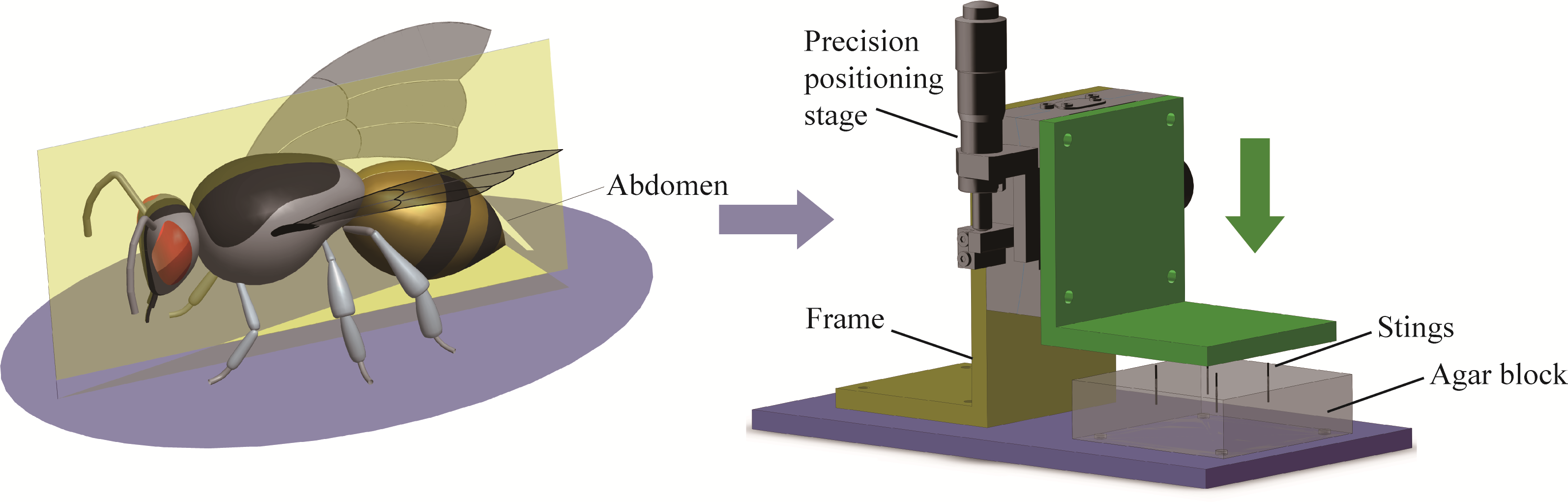  (a)Sample of the honey bee (b) Setup of penetration. |
| --- |
| **Figure S1.** Principle of experiment |

Figure S2(a) shows 5 fresh samples of stings of the worker bees. Then the sting samples are arranged vertically to the surface of the substrate, shown in Figure S2(b).

| 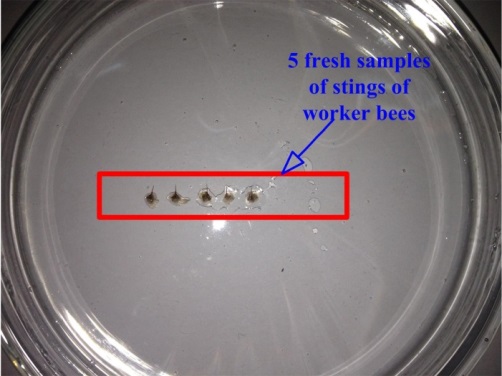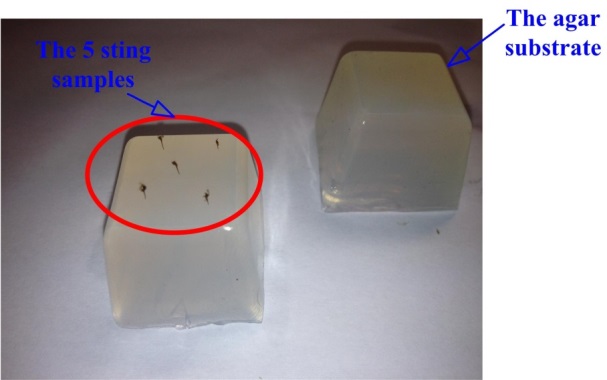  (a)Fresh sting samples (b)Sting samples and agar substrate |
| --- |
| **Figure S2.** Samples of the worker bees’ stings |

To test the behavior of penetration, the precision positioner is applied to insert the stings into the substrates (Figure S3). Note that the cross sections of the sting samples are observed before and after penetration. And the inserted length of the sting samples is 6*mm*.

| **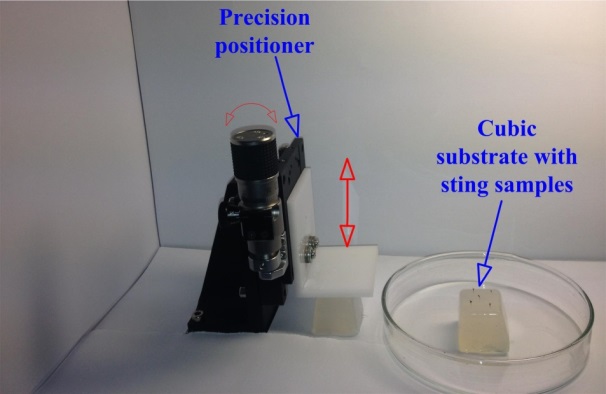** |
| --- |
| **Figure S3.** The precision positioner and the substrate |

The positioning mark is used to control the position of sting samples (Figure S4).

| **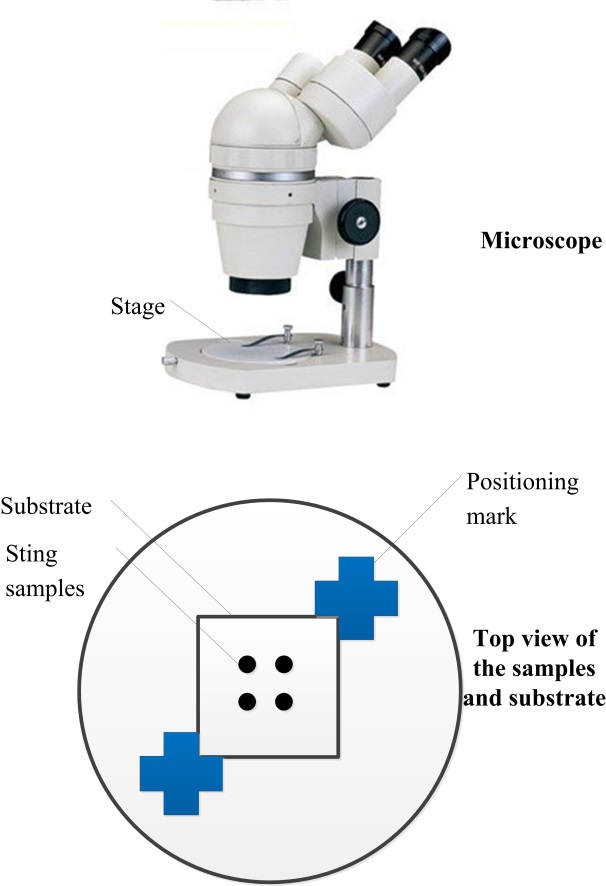** |
| --- |
| **Figure S4.**  Location of the substrate. |

**2. The measurement technique of the rotation angles**

Processed by the Canny operator, the morphology of the cross section under the microscope are shown in Figure S5.

| 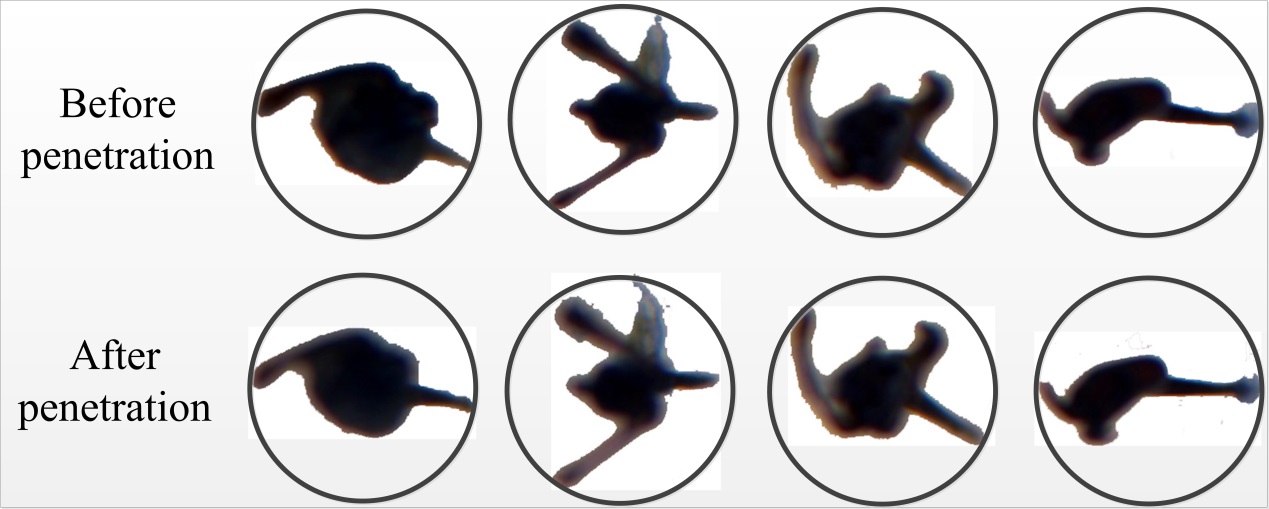  (A)Cross sections before penetration  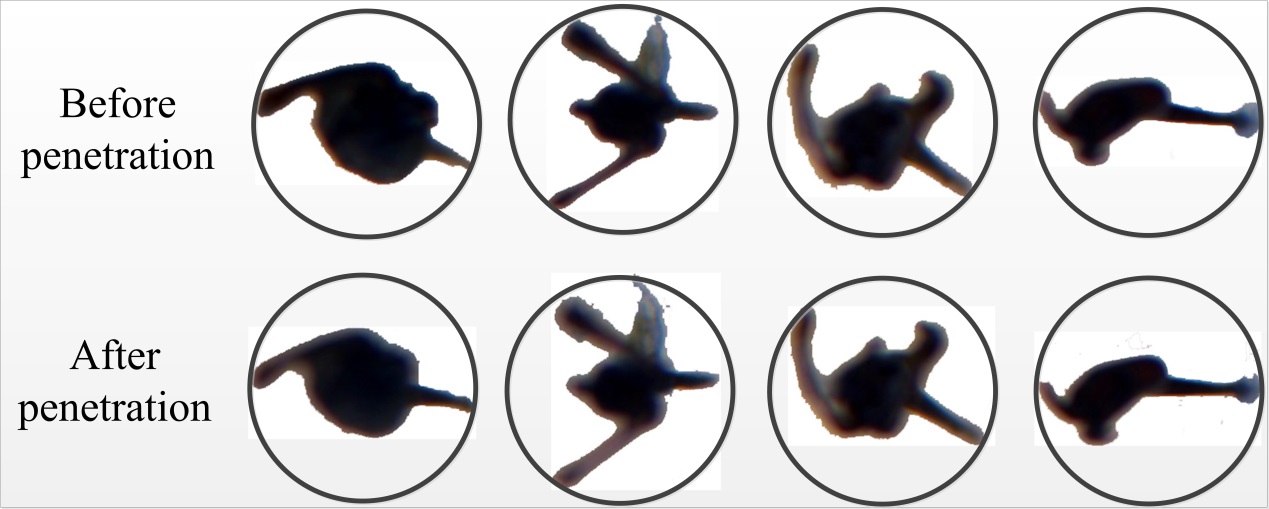  (B) Cross sections after penetration |
| --- |
| **Figure S5.** Morphology of the cross section. |

The rotation angles are shown in Figure S6, also presented in the main document.

| **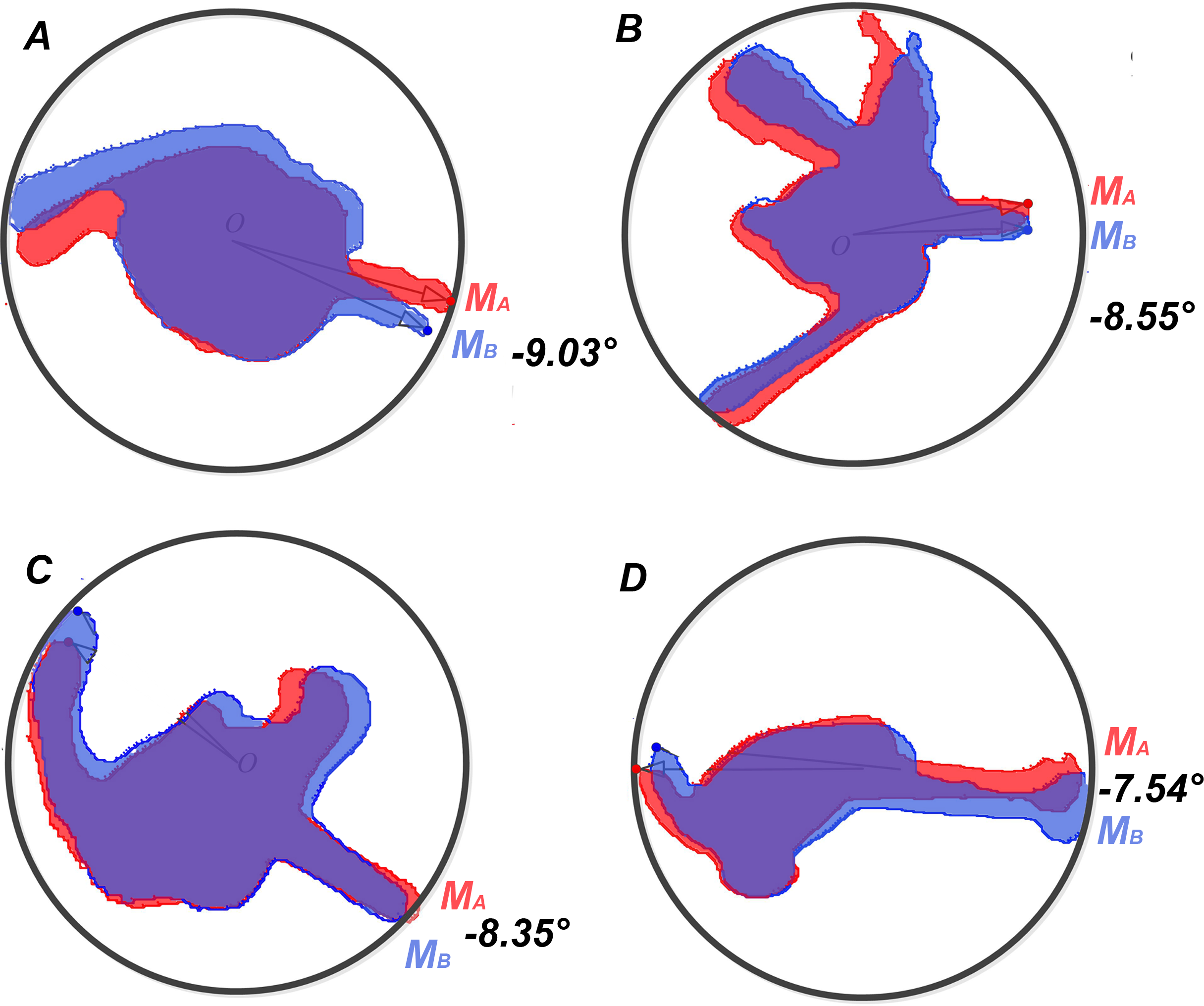** |
| --- |
| **Figure S6.** Rotation angles of the sting shaft. |

Table S1 shows the experimental results of helical penetration by using the agar, silica gel, soft rubber and paraffin.

**Table S1**: The experimental results from the observation of the rotational angles

1. Material: Agar (A-anti-clockwise; C-clockwise)

| **No.** | **Angle/degree** | **Inserted length/mm** | **Hardness/Pa** |
| --- | --- | --- | --- |
| **Sample_1** | 9.03(C) | 6.00 | 370 |
| **Sample_2** | 8.89(C) |
| **Sample_3** | 9.15(C) |
| **Sample_4** | 8.95(C) |
| **Sample_5** | 8.99(C) |
| **Average** | **9.002(C)** |

(b) Material: Silica gel (A-anti-clockwise; C-clockwise)

| **No.** | **Angle/degree** | **Inserted length/mm** | **Hardness/Pa** |
| --- | --- | --- | --- |
| **Sample_6** | 8.55(C) | 6.00 | 800 |
| **Sample_7** | 8.55(C) |
| **Sample_8** | 8.57(C) |
| **Sample_9** | 8.59(C) |
| **Sample_10** | 8.56(C) |
| **Average** | **8.564(C)** |

(c) Material: Soft rubber (A-anti-clockwise; C-clockwise)

| No. | Angle/degree | Inserted length/mm | Hardness |
| --- | --- | --- | --- |
| **Sample_11** | 8.35(C) | 6.00 | 1000 |
| **Sample_12** | 8.33(C) |
| **Sample_13** | 8.37(C) |
| **Sample_14** | 8.35(C) |
| **Sample_15** | 8.37(C) |
| **Average** | **8.354(C)** |

(d) Material: Paraffin (A-anti-clockwise; C-clockwise)

| No. | Angle/degree | Inserted length/mm | Hardness |
| --- | --- | --- | --- |
| **Sample_16** | 7.54(C) | 6.00 | 1200 |
| **Sample_17** | 7.39(C) |
| **Sample_18** | 7.38(C) |
| **Sample_19** | 7.51(C) |
| **Sample_20** | 7.50(C) |
| **Average** | **7.464(C)** |
